# Minimally invasive plate osteosynthesis vs conventional fixation techniques for surgically treated humeral shaft fractures: a meta-analysis

**DOI:** 10.1186/s13018-016-0394-x

**Published:** 2016-05-11

**Authors:** Xuqi Hu, Siqi Xu, Huigen Lu, Bao Chen, Xiao Zhou, Xiaojun He, Jiaping Dai, Zhongwei Zhang, Suiliang Gong

**Affiliations:** Department of Orthopaedics, the Second Affiliated Hospital of Jiaxing University, 1518 Huancheng North Road, Jiaxing, China; Department of Clinical Laboratory, the Second Affiliated Hospital of Jiaxing University, 1518 Huancheng North Road, Jiaxing, China

**Keywords:** Minimally invasive plate osteosynthesis, Open reduction with plate osteosynthesis, Intramedullary nail, Humeral shaft fracture, Meta-analysis

## Abstract

**Background:**

In this study, we performed a meta-analysis to identify whether minimally invasive plate osteosynthesis (MIPO) was superior to conventional fixation techniques (CFT) for treating humeral shaft fractures.

**Methods:**

A systematic literature search was conducted up to February 2016 in ScienceDirect, Springer, MEDLINE, and PubMed databases for relevant papers that compared the outcomes of MIPO with CFT, such as open reduction with plate osteosynthesis (ORPO) and intramedullary nail (IMN) for treating humeral shaft fractures. Meta-analysis was performed with Review Manager 5.0 software.

**Results:**

According to the search strategy, eight studies that covered 391 patients were enrolled, including four randomized controlled trials (RCTs), two prospective cohort trials, and two retrospective cohort trials. Our meta-analysis did not detect any significant difference between MIPO and CFT (IMN and ORPO) in terms of operative time, fracture union rate, and fracture union time. However, MIPO has a less rate of complications and iatrogenic radial nerve palsy than that of ORPO and higher adjacent joint function scores than those of IMN (*p* < 0.05).

**Conclusions:**

Based on the present evidence, this meta-analysis suggested that MIPO was a better choice for treating humeral shaft fractures than CFT. However, more high-quality randomized trials are still needed to further confirm this conclusion in the future.

## Background

Fractures of humeral shaft are common injuries, which make up 1 to 3 % of all fractures [1–5]. Historically, nonoperative treatment has been widely used for these injuries. However, a high rate of nonunion was reported in humeral shaft fracture patients with functional bracing [6, 7]. Therefore, many orthopedic surgeons tend to prefer operative treatment for humeral shaft fractures.

Three main operative techniques have been developed for treating displaced humeral shaft fractures. Intramedullary nail and plate are the conventionally used surgical methods [5, 8]. Currently, open reduction and plate fixation remains to be the golden standard for humeral shaft fractures [9, 10]. Recently, minimally invasive plate osteosynthesis (MIPO) techniques with encouraging results in humeral shaft fracture patients have been reported [11–14]. This technique has advantages of less soft tissue dissection, a high rate of union, low risks of infection, and no need for radial nerve exposure [15]. It seems to imply that MIPO is superior to conventional fixation techniques (CFT), such as open reduction with plate osteosynthesis (ORPO) and intramedullary nail (IMN).

Recently, several randomized controlled trials (RCTs) and comparative clinical studies have been conducted to compare MIPO with CFT for treating humeral shaft fractures. In this study, we performed a meta-analysis to identify whether MIPO was superior to CFT for treating humeral shaft fractures.

## Methods

### Search strategy

Since there were only a small amount of relevant RCTs in the literature, observational studies were also included. A systematic literature search was conducted up to February 2016 in ScienceDirect, Springer, MEDLINE, and PubMed databases. We screened the title and abstract with key words as follows: “minimally invasive plate osteosynthesis” or “MIPO”, “plate” or “plating”, “intramedullary nail” or “intramedullary pin”, and “humeral shaft fracture” or “fracture of humeral shaft”. In addition, references of the selected articles and relevant review papers were also searched. Unpublished data were not reviewed. The language of articles was limited to English.

### Inclusion and exclusion criteria

The following eligibility criteria were applied in selecting articles: (1) RCTs or observational studies that compared the clinical and/or radiological outcomes of MIPO with CFT for treating humeral shaft fractures; (2) totally followed patients had to be more than 30; and (3) skeletally mature patients. The exclusion criteria included the following: (1) a pathologic fracture; (2) studies that did not report the outcome of interest; and (3) repeated studies or reviews. Two people independently performed the selection of studies. Any disagreement between the reviewers was resolved by consensus with a third reviewer.

### Data extraction

Two reviewers extracted data independently based on the following categories: (1) basic characteristics, such as study design, published year, study population characteristics, and humeral shaft fracture type; (2) primary outcomes, consisting of postoperative clinical function evaluated by the University of California, Los Angeles (UCLA) Shoulder Scale [16] and Mayo Elbow performance score (MEPS) [17]; and (3) secondary outcomes, such as complications and iatrogenic radial nerve palsy, operative time, radiation exposure time, and fracture union time. Any disagreement between the reviewers was resolved by consensus with a third reviewer.

### Risk of bias assessment

To assess the risk of bias of the included RCTs, the *Cochrane Handbook for Systematic Reviews of Interventions* was applied. The risk of bias of the included observational studies was evaluated with the Newcastle–Ottawa Scale, and the trials with a total score over 5 were considered to be of high quality [18].

### Statistical analysis

Meta-analysis was performed using Review Manager 5.0 software (Cochrane Collaboration, Oxford, UK). Weighted mean difference (WMD) or standard mean difference (SMD) was calculated for continuous outcomes and risk ratios (RR) for binary outcomes, along with 95 % confidence intervals (CIs). The level of significance was set at *p* < 0.05. Heterogeneity was evaluated using the *χ*^2^ test and *I*^2^ statistics. (Heterogeneity was detected when *p* < 0.10 or *I*^2^ > 50 %.) Fixed-effects models were applied unless statistical heterogeneity was significant, in which case random-effects models were used. Standard deviation (SD) was estimated according to the method described by the *Cochrane Handbook for Systematic Reviews of Interventions* when it was not available. In this paper, CFT was consisted of ORPO and IMN. Then, we conducted subgroup analyses based on the two kinds of CFT (ORPO subgroup and IMN subgroup).

## Results

### Literature search

**Fig. 1:**
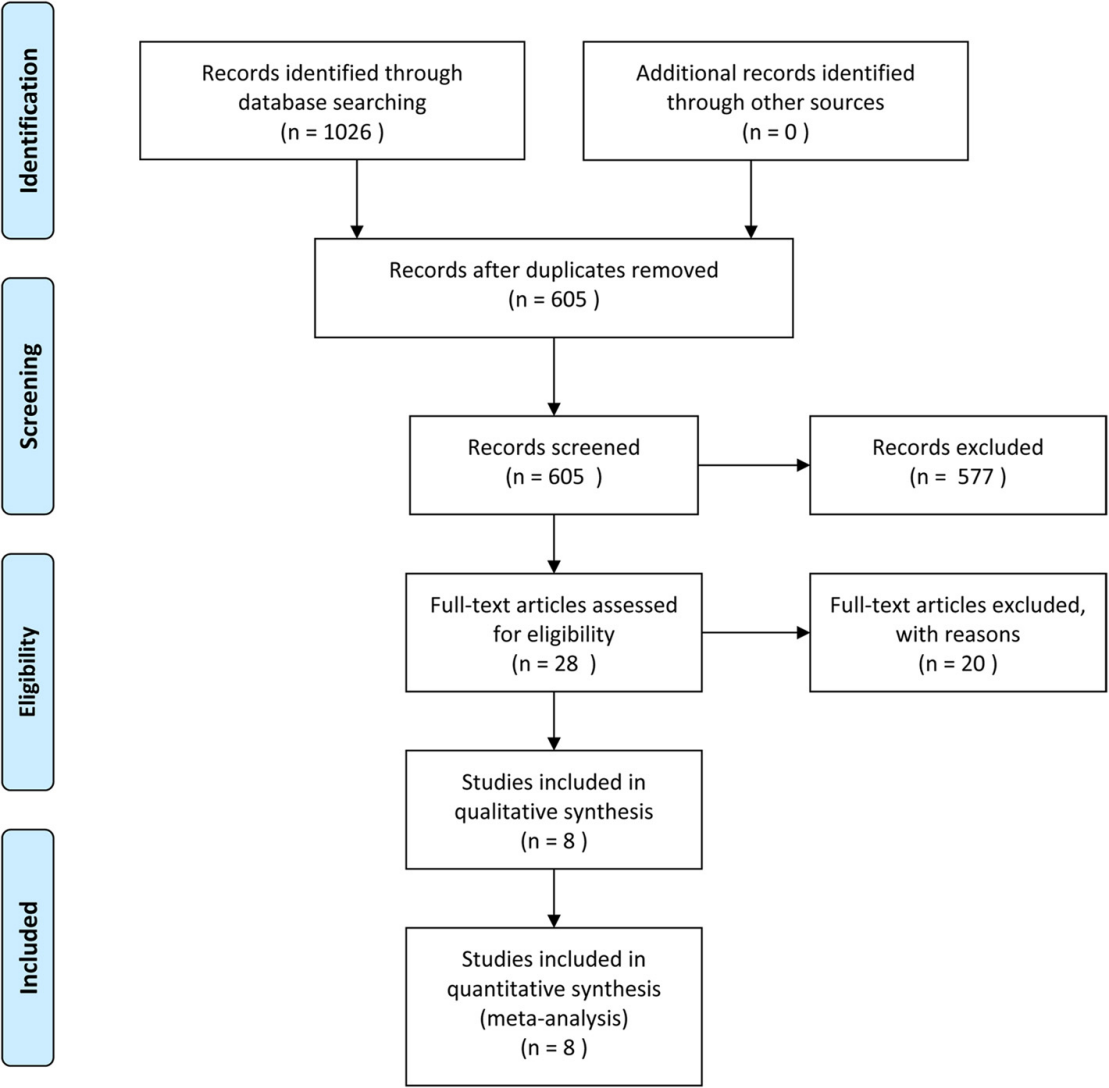
Flowchart of the articles included in this meta-analysis

According to the search strategy, 1026 articles were identified initially, of which 421 were screened after removal of duplicated records. Then, 577 studies were excluded due to inappropriate topics. The full text of the remaining 28 papers were obtained and assessed for eligibility. Twenty of them were further removed according to predefined inclusion/exclusion criteria. Finally, four randomized controlled trials, two prospective cohort studies, and two retrospective cohort trials were enrolled in this study (Fig. 1).

### Study characteristics

The basic information of the eight included studies is shown in Table 1. These studies were published from 2010 to 2015, including four RCTs [19–22], two prospective cohort trials [23, 24], and two retrospective cohort trials [25, 26]. Patients with open fracture or radial nerve injury were excluded in five trials [19, 22, 24–26]. Two studies included patients with radial nerve injury but excluded Gustilo-Anderson [27] III open fractures [21, 23]. One paper excluded Gustilo-Anderson open fractures [27] classified IIIb or IIIa and patients with radial nerve injury [20]. A total of 391 patients were evaluated which covered 196 patients in the MIPO group and 195 patients in the CFT group. There were three papers comparing MIPO with IMN [19, 20, 26] and five papers comparing MIPO with ORPO [21–25]. Among 196 patients treated by MIPO, 60 patients were compared with 61 patients treated by IMN and 136 patients were compared with 134 patients treated by ORPO.

**Table 1:** Characteristics of the eight included trials

Characteristic	An	An	Oh	Lian	Benegas	Wang	Kim	Esmailiejah
Publication year	2010	2012	2012	2013	2014	2015	2015	2015
Study design	Retro	Retro	Pro	RCT	RCT	Pro	RCT	RCT
No. of enrolled patients (MIPO vs CFT)	17:16	15:19	29:30	24:23	21:19	26:27	36:36	33:35
No. of followed patients (MIPO vs CFT)	17:16	15:19	29:30	24:23	21:19	22:23	36:32	32:33
Follow-up rate (%; MIPO vs CFT)	100:100	100:100	100:100	100:100	100:100	84.6:85.2	100:88.9	97.0:94.3
Mean follow-up time (months; MIPO vs CFT)	25.94:32.88	24.2:20.5	18:22	14:15	12:12	12:12	15	N/A
Mean age (years; MIPO vs CFT)	37.59:36.93	34.4:39.6	39.6:42	38.8:37.6	44.8:38.4	39.3:35.7	40.6:44.4	33.4:34.6
Gender (% male; MIPO vs CFT)	70.6:56.3	73.3:63.2	55.2:53.3	62.5:69.6	57.1:73.7	63.6:69.6	52.8:56.3	75:72.7
Fracture location (proximal/middle/distal; MIPO vs CFT)	0/8/9:0/9/7	0/15/0:0/19/0	6/18/5:5/20/5	0/24/0:0/23/0	N/A	4/13/5:2/15/6	4/21/11:4/16/12	N/A
Fracture classification* (A/B/C; MIPO vs CFT)	N/A	6/7/2:10/8/1	11/11/7:15/8/7	9/9/5:8/12/2	12/7/2:9/4/6	5/8/9:5/12/6	19/17/0:21/11/0	10/9/13:12/10/11
Intervention (MIPO vs CFT)	DCP vs DCP	DCP vs IMN	LCP vs LCP	DCP vs IMN	DCP vs IMN	LCP vs LCP	LCP vs LCP	DCP vs DCP

*RCT* randomized controlled trial, *Retro* retrospective cohort study, *Pro* prospective cohort study, *MIPO* minimally invasive plate osteosynthesis, *CFT* conventional fixation techniques, *N/A* not available, *DCP* dynamic compression plate, *LCP* locking compression plate, *IMN* intramedullary nail. * AO/OTA classification

### Risk of bias assessment

The risk of bias assessment of the four included RCTs is shown in Table 2. All the RCTs described adequate methods of random sequence generation [19–22]. However, only one paper described allocation concealment [20]. All trials were reported as high risk since it was impossible to perform blinding of participants and personnel. We regarded these studies as low risk of incomplete outcome data addressed because only seven patients lost to follow-up. All of the included RCTs provided the outcomes in detail. The risk of bias of the included cohort trials evaluated with the Newcastle–Ottawa Scale is demonstrated in Table 3. All these cohort trials had a score over 5.

**Table 2:** Risk of bias assessment of randomized controlled trials

Risk of bias assessment	Lian 2013 [19]	Benegas 2014 [20]	Kim 2015 [21]	Esmailiejah 2015 [22]
Random sequence generation	Low	Low	Low	Low
Allocation concealment	Unclear	Low	Unclear	Unclear
Blinding of participants and personnel	High	High	High	High
Blinding of outcome assessment	Unclear	Low	Low	Unclear
Incomplete outcome data addressed	Low	Low	Low	Low
Selective reporting	Low	Low	Low	Low
Other bias	Unclear	Unclear	Unclear	Unclear

**Table 3:** The Newcastle–Ottawa scale score of case control study/cohort study

Study ID	Selection	Comparability	Outcome/exposure	Total score
An 2010 [25]	3	2	3	8
An 2012	3	1	3	7
Oh 2012 [23]	4	2	2	8
Wang 2015 [24]	4	2	3	9

### Postoperative clinical function (UCLA, MEPS)

**Fig. 2:**
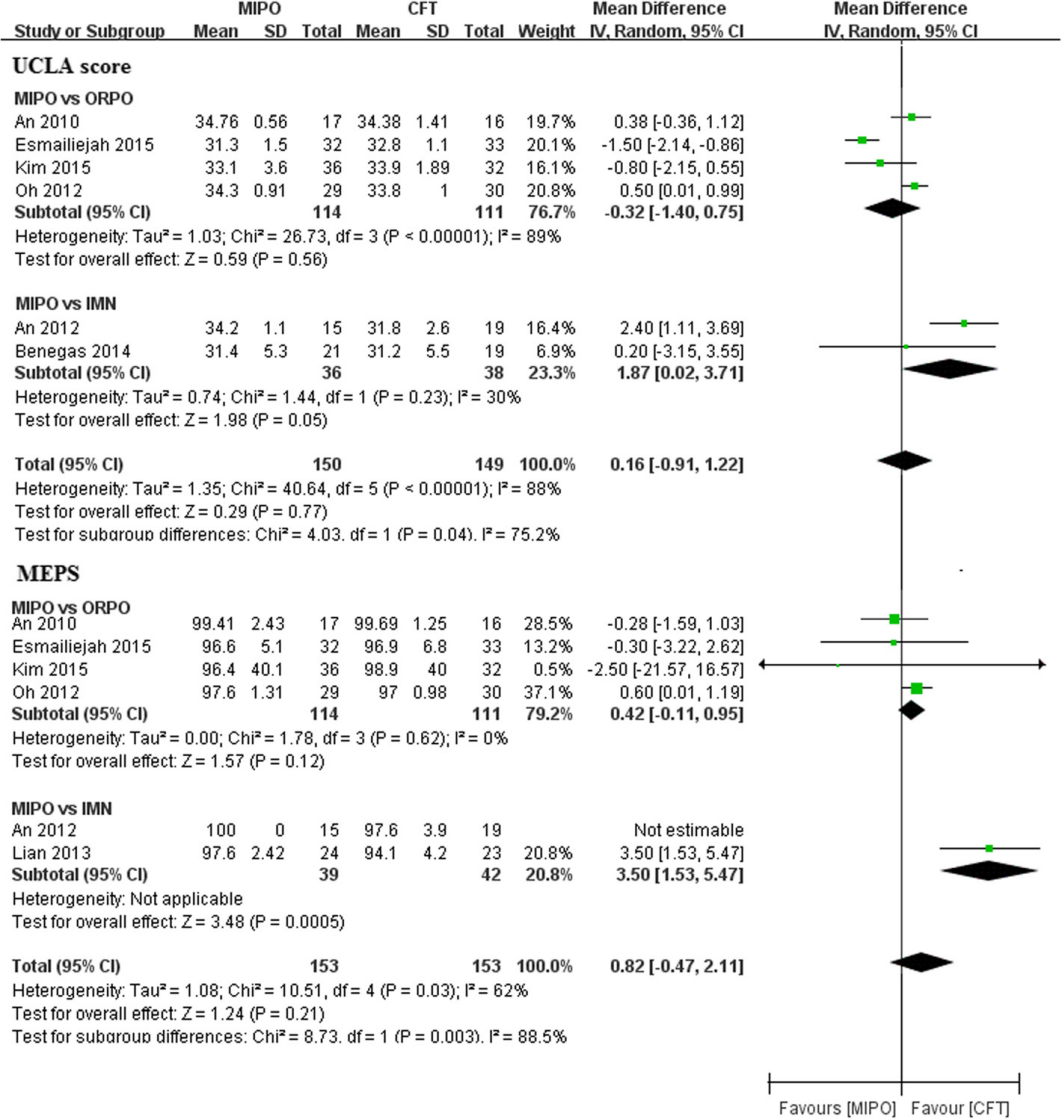
Forest plot illustrating the UCLA score and MEPS of meta-analysis MIPO with CFT (ORPO and IMN) in humeral shaft fractures

Shoulder function was assessed using the UCLA score in six studies [20–23, 25, 26]. Among these studies, four papers were correlated to the ORPO subgroup [21–23, 25] and others were correlated to the IMN subgroup [20, 26]. Meta-analysis showed no significant difference in the ORPO subgroup (WMD = −0.32, 95 % CI −1.40–0.75, *p* = 0.56; *I*^2^ = 89 %; *p* < 0.01) or the IMN subgroup (WMD = 1.87, 95 % CI 0.02–3.71, *p* = 0.05; *I*^2^ = 30 %; *p* = 0.23). Pool analysis of all studies also did not reveal any significant difference between the MIPO group and the CFT group for the UCLA score (WMD = 0.16, 95 % CI −0.91–1.22, *p* = 0.77; *I*^2^ = 88 %; *p* < 0.01) (Fig. 2).

MEPS was applied to evaluate elbow function in six studies [19, 21–23, 25, 26]. Among these studies, four papers were correlated to the ORPO subgroup [21–23, 25] and the other two papers were correlated to the IMN subgroup [19, 26]. Meta-analysis showed that MEPS was significantly higher in the MIPO group than in the IMN group (WMD = 3.5, 95 % CI 1.53–5.47; *p* = 0.0005). There was no significant difference between the two arms either in the ORPO subgroup (WMD = 0.42, 95 % CI −0.11–0.95, *p* = 0.12; *I*^2^ = 0 %; *p* = 0.62) or in the total studies (WMD = 0.82, 95 % CI −0.47–2.11, *p* = 0.21; *I*^2^ = 62 %; *p* = 0.03) (Fig. 2).

### Complications and iatrogenic radial nerve palsy

**Fig. 3:**
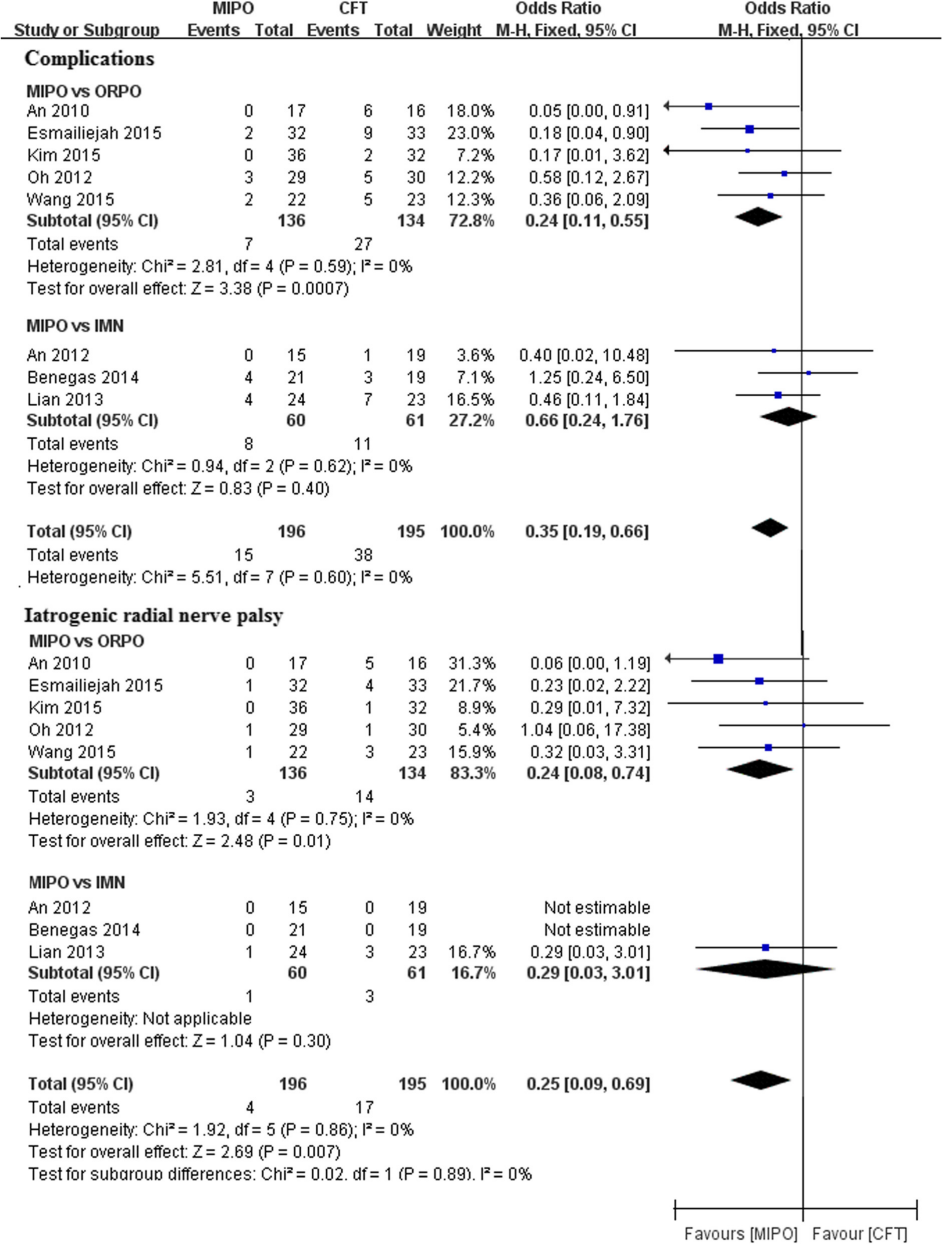
Forest plot illustrating complications and iatrogenic radial nerve palsy of meta-analysis MIPO with CFT (ORPO and IMN) in humeral shaft fractures

All of the included studies reported the outcome of complications. The pooled data demonstrated a higher complication rate in the CFT group than in the MIPO group (RR = 0.35, 95 % CI 0.19–0.66, *p* = 0.001; *I*^2^ = 0 %; *p* = 0.62). Subgroup analysis showed that the complication rate in ORPO was significantly higher than that in MIPO (RR = 0.24, 95 % CI 0.11–0.55, *p* = 0.0007; *I*^2^ = 0 %; *p* = 0.59). However, no significant difference was detected in the IMN subgroup (RR = 0.66, 95 % CI 0.24–1.76, *p* = 0.40; *I*^2^ = 0 %; *p* = 0.62) (Fig. 3).

Iatrogenic radial nerve palsy was also available in all of the included papers. Meta-analysis showed that the rate of iatrogenic radial nerve palsy was significantly higher in the CFT group than that in the MIPO group (RR = 0.25, 95 % CI 0.09–0.69, *p* = 0.007; *I*^2^ = 0 %; *p* = 0.86). Subgroup analysis also detected a significant difference between MIPO and ORPO (RR = 0.24, 95 % CI 0.08–0.74, *p* = 0.01; *I*^2^ = 0 %; *p* = 0.75). However, subgroup analysis did not reveal any significant difference between MIPO and IMN (RR = 0.29, 95 % CI 0.03–3.01, *p* = 0.30) (Fig. 3).

### Fracture union rate and union time

**Fig. 4:**
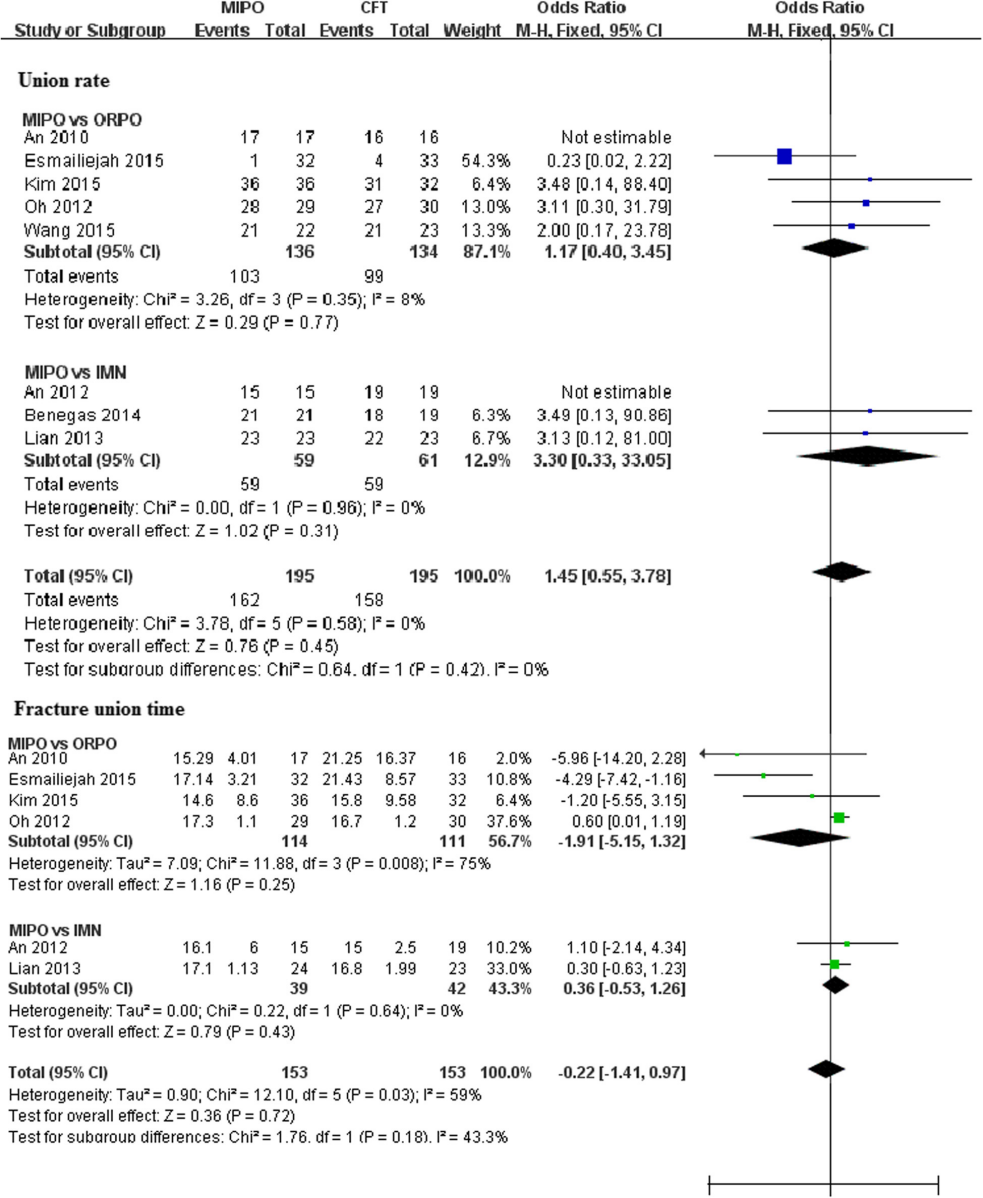
Forest plot illustrating union rate and union time of meta-analysis MIPO with CFT (ORPO and IMN) in humeral shaft fractures

Data regarding fracture union was reported in all of the included studies. No significant difference was detected either in the ORPO subgroup (RR = 1.17, 95 % CI 0.40–3.45, *p* = 0.77; *I*^2^ = 8 %; *p* = 0.35) or in the IMN subgroup (RR = 3.30, 95 % CI 0.33–33.05, *p* = 0.31; *I*^2^ = 0 %; *p* = 0.96). Moreover, the pooled estimate also showed no significant difference between the MIPO group and the CFT group (RR = 1.45, 95 % CI 0.55–3.78, *p* = 0.45; *I*^2^ = 0 %; *p* = 0.58) (Fig. 4).

Fracture union time was available in six trials [19, 21–23, 25, 26]. Four papers were correlated to the ORPO subgroup [21–23, 25], and the other two were correlated to the IMN subgroup [19, 26]. When all studies were considered, meta-analysis did not find any significant difference between the MIPO and CFT groups (WMD = −0.22, 95 % CI −0.41–0.97, *p* = 0.72; *I*^2^ = 59 %; *p* = 0.03). Subgroup analysis also did not detect any significant difference in the ORPO subgroup (WMD = −1.91, 95 % CI −5.15–1.32 *p* = 0.25; *I*^2^ = 75 %; *p* = 0.008) or the IMN subgroup (WMD = 0.36, 95 % CI −0.53–1.26, *p* = 0.43; *I*^2^ = 0 %; *p* = 0.64) (Fig. 4).

### Operative time and radiation exposure time

**Fig. 5:**
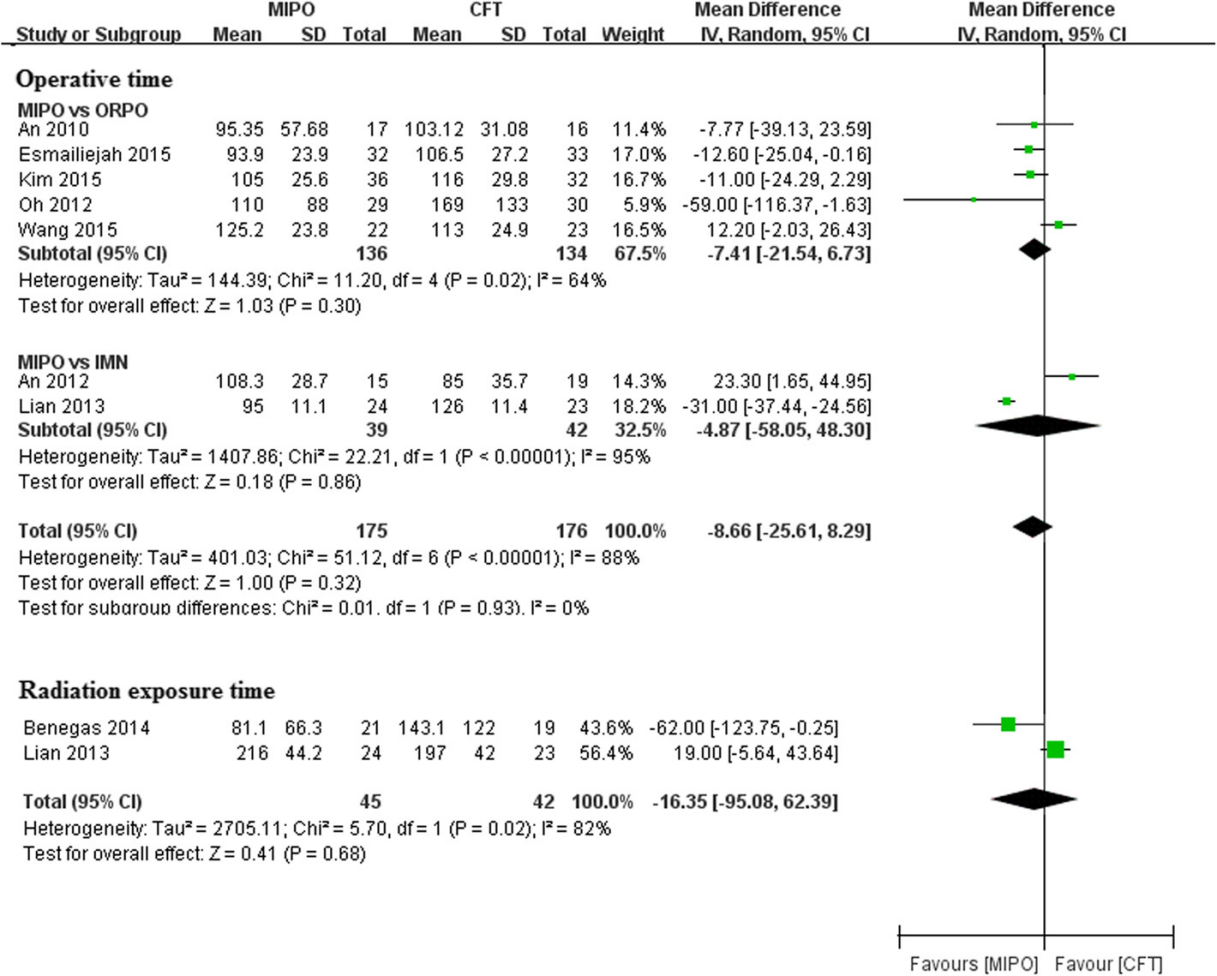
Forest plot illustrating operative time and radiation exposure time of meta-analysis MIPO with CFT (ORPO and IMN) in humeral shaft fractures

Seven studies reported the data of operative time [19, 21–26]. Subgroup analysis also did not find any significant difference in the ORPO subgroup (WMD = −7.41, 95 % CI −21.54–6.73, *p* = 0.30; *I*^2^ = 64 %; *p* = 0.02) or in the IMN subgroup (WMD = −4.87, 95 % CI −58.05–48.30, *p* = 0.86; *I*^2^ = 95 %; *p* < 0.01). Meta-analysis of total data revealed that the difference was not statistically significant between the MIPO group and the CFT group (WMD = −8.66, 95 % CI −25.61–8.29, *p* = 0.32; *I*^2^ = 88 %; *p* < 0.01) (Fig. 5).

Since fluoroscopy was not applied in the ORPO subgroup, two paper correlated to the IMN group provided the data of radiation exposure time during surgery [19, 20]. Pooled analysis did not detect any significant difference between IMN and MIPO (WMD = −16.35, 95 % CI −95.08–62.39, *p* = 0.68; *I*^2^ = 82 %; *p* = 0.02) (Fig. 5).

## Discussion

Although ORPO remains the main standard of operative fixation for humeral shaft fractures, this technique has certain disadvantages of extensive incision, increased incidence of iatrogenic radial nerve palsy, high risk of infection, and violation of the fracture site blood supply [3, 5, 10]. Therefore, in consideration of ORPO, IMN, and MIPO, no consensus has been reached on the optimal technique for humeral shaft fractures. Our meta-analysis did not detect any significant difference between MIPO and CFT (IMN and ORPO) in terms of operative time, fracture union rate, and fracture union time. In other words, compared with CFT, MIPO did not have the advantages of a higher fracture union rate or earlier union time.

Due to biomechanical characteristics and load-sharing capacity of the implant, IMN has achieved satisfying results in humeral shaft fractures. However, shoulder problems after IMN surgery also attract numerous orthopedic surgeons’ attention [5, 28, 29]. Injuries of the rotator cuff and impingement caused by prominent nail end are thought to be the main reasons for shoulder disfunction. Although subgroup analyses did not demonstrate any significant difference in the UCLA score between MIPO and IMN (*p* = 0.05), the result might be changed provided that the sample size was increased. Retrograde IMN approach is usually adopted to prevent shoulder problems. However, this benefit is obtained at the cost of supracondylar fracture and elbow problems [30].

Three papers in the IMN subgroup estimated the elbow function by the Broberg-Morrey score or MEPS. There was no significant difference in elbow function between IMN and MIPO in An or Benegas’s studies [20, 26], where only antegrade IMN approach was used. Interestingly, in Lian’s trial [19], where antegrade or retrograde IMN approach was applied, the MEPS in MIPO was significantly higher than that in IMN. In contrast, no significant difference was revealed in the UCLA score or MEPS between MIPO and ORPO.

Regarding the safety of therapies, the total complication rate of MIPO was 5.14 % (7/136) while the total complication rate of ORPO was 20.15 % (27/134) in the ORPO subgroup. Subgroup analyses detected a significant difference in the complication rate between MIPO and ORPO (*p* < 0.01). However, no significant difference was observed in the complication rate between MIPO and IMN. During the ORPO surgery, the radial nerve was dissected and the fracture site was exposed which resulted in disruption of periosteal blood supply. It was not surprising that the main complications reported in the ORPO subgroup were iatrogenic radial nerve palsy, nonunion, and infection.

Since injury of the radial nerve is a disastrous intraoperative complication in humeral shaft fractures, iatrogenic radial nerve palsy was evaluated separately in our meta-analysis. MIPO, as previously noted, has the advantage of no need for radial nerve exposure [15]. The total rate of iatrogenic radial nerve palsy in MIPO was 2.20 % (3/136) in five studies, which was significantly lower than that in ORPO (10.45 %, 14/134, *p* = 0.01). However, rich anatomical knowledge and long learning curve is required for MIPO. Otherwise, MIPO may lead to a high rate of radial nerve palsy or nonunion. Since the radial nerve was not dissected in IMN surgery either, subgroup analysis did not reveal any significant difference in iatrogenic radial nerve palsy between MIPO and IMN (*p* = 0.30).

There are some limitations in this study. First of all, only eight articles covering 391 patients were included in this meta-analysis, of which only four were RCTs. This may weaken the strength of the evidence of this paper. Secondly, there were some confounding factors such as confirmation of complication. Pooling such data may lead to bias. At last, some baseline characteristics were different among the trials. There are various internal implants in the included studies, such as dynamic compression plate, locking compression plate, reamed IMN, or undreamed IMN. This may have potential effects on clinical and radiological outcomes.

## Conclusions

In summary, based on the present evidence, MIPO is a better choice for treating humeral shaft fractures than CFT, though there is no significant difference between MIPO and CFT in terms of operative time, fracture union rate, and fracture union time. MIPO has a less rate of complications and iatrogenic radial nerve palsy than that of ORPO and higher adjacent joint function scores than those of IMN. However, more high-quality randomized trials are still needed to further confirm this conclusion in the future.
